# Separation of surface oxide from bulk Ni by selective Ni 3*p* photoelectron spectroscopy for chemical analysis in coincidence with Ni *M*-edge Auger electrons

**DOI:** 10.1038/s41598-021-96108-x

**Published:** 2021-08-16

**Authors:** Artur Born, Fredrik O. L. Johansson, Torsten Leitner, Danilo Kühn, Andreas Lindblad, Nils Mårtensson, Alexander Föhlisch

**Affiliations:** 1 Uppsala-Berlin Joint Laboratory on Next Generation Photoelectron Spectroscopy, Albert-Einstein-Str. 15, 12489 Berlin, Germany; 2 Institute Methods and Instrumentation for Synchrotron Radiation Research PS-ISRR, Helmholtz-Zentrum Berlin für Materialien und Energie, Albert-Einstein-Straße 15, 12489 Berlin, Germany; 3 Institut für Physik und Astronomie, Universität Potsdam, Karl-Liebknecht-Strasse 24-25, 14476 Potsdam, Germany; 4 Department of Physics and Astronomy, Molecular and Condensed Matter Physics, Uppsala University, P.O. Box 256, 751 05 Uppsala, Sweden

**Keywords:** Electronic properties and materials, Surfaces, interfaces and thin films, Condensed-matter physics, Techniques and instrumentation

## Abstract

The chemical shift of core level binding energies makes electron spectroscopy for chemical analysis (ESCA) a workhorse analytical tool for science and industry. For some elements, close lying and overlapping spectral features within the natural life time broadening restrict applications. We establish how the core level binding energy chemical shift can be picked up experimentally by the additional selectivity through Auger electron photoelectron coincidence spectroscopy (APECS). Coincident measurement of Ni 3*p* photoemission with different *MVV* Auger regions from specific decay channels, narrows the 3*p* core-levels to a width of 1.2 eV, resolves the spin–orbit splitting of 1.6 eV and determines the chemical shift of Ni 3*p* levels of a Ni(111) single crystal and its oxidized surface layer to 0.6 eV.

## Introduction

Electron spectroscopy for chemical analysis (ESCA) is a powerful analytical tool^1^. For overlapping spectral lines the natural life time broadening hampers for some elements the chemical shift determination in classical ESCA. For example, detailed electronic structure determination and chemical state analysis for Nickel based materials at the spin orbit split Ni $3p_{3/2}$ and $3p_{1/2}$ (*M*-edge) core levels have been limited by this condition, despite the fact that they provide important information about the nature of correlation effects^2–7^. Thus, photoelectron spectroscopy (PES) experiments typically focus on either the valence band or the 2*p* core-levels, where the satellites are well known. The 3*p* core-levels however are less explored, since the $3p_{1/2}$ and $3p_{3/2}$ spectral ESCA features massively overlap, due to the small spin–orbit splitting and large lifetime broadening. Especially in Ni, the reported widths and positions of the 3*p* core-levels show large variations depending on the experiment and analysis^8–11^. Also the core-hole decay in 3*d* metals is excessively studied using Auger electron spectroscopy (AES) and Auger electron photoelectron coincidence spectroscopy (APECS) giving access to two-hole final states^12–14^. Similar to ESCA in case of Ni AES, most work is devoted to the *LVV* Auger decay^2,7,15,16^ and only few investigations have been done studying the *MVV* or *MVV* super Coster–Kronig decay^8,17^.

**Figure 1:**
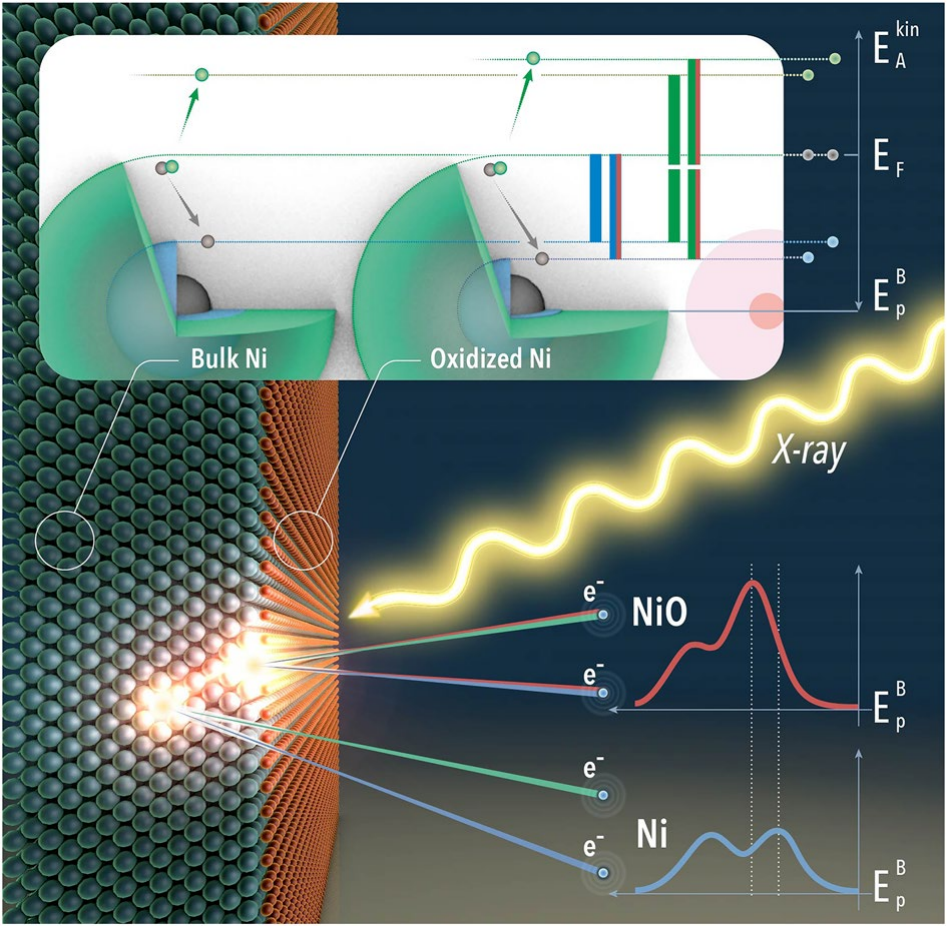
APECS measurement scheme on a Ni single crystal with an oxidized surface layer. The X-ray beam ionizes atoms either in the bulk or oxidized on the surface producing photoelectrons and corresponding Auger electrons. Comparison of the photoelectron binding energy leads to a chemical shift caused by the different chemical environments, which can be resolved measuring the photoelectrons (blue) in coincidence with the Auger electrons (green). The insert shows the energy scheme of the bulk and oxidized surface components. The binding energy of the 3*p* levels is shown in blue. The corresponding Auger decay is indicated in green. For the oxidized Ni atoms the 3*p* levels are shifted towards higher binding energies resulting in higher Auger electron kinetic energy. The electrons and the energies from chemical shifted atoms are additionally marked by a red line. The illustration was created with Adobe Photoshop^18^, Adobe Illustrator^19^ and Blender^20^.

Adsorption of $\hbox {N}_2$, $\hbox {O}_2$, C or H on Ni surfaces was extensively studied by the electron spectroscopy community, due to a wide range of structural and electronic modifications^21–24^. As for the pure Ni, most spectroscopic studies concentrated on the *L* edge or the valence band. This is due to the distinct additional features and chemical shifts corresponding to the modification of the chemical environment. Especially NiO stands out, since some transition metal oxides are considered as prototypical Mott-insulators^25,26^. Thus, the oxidation process of the surface, the electronic structure as well as the chemical shifts were well investigated utilizing ESCA and AES, focusing on the 2*p* core-levels, the valence band and the *LVV* Auger decay^27–29^.

Despite the large interest in NiO, the 3*p* levels are scarcely investigated. A. M. Venezia and C. M. Loxton^30^ reported a chemical shift of the Ni 3*p* levels about 1.5 eV towards higher binding energies in the $\hbox {Ni}_3$Al alloy, while N. S. McIntyre^11^ reported a shift of about 1 eV in NiO and 1.7 eV in Ni(OH)$_2$, both utilizing conventional PES.

APECS is especially well suited to study oxidized surfaces, since on the one hand it provides an extraordinary surface sensitivity, and on the other hand it allows to disentangle features corresponding to different two-hole final states^31^. Other studies demonstrated the capabilities of the method and could separate bulk and surface components on Ta and TaC^32,33^ or study the double photoemission or 3*p* core-hole relaxation on Cu^34–36^. Also coincidence studies on oxidized metals and semiconductors were performed, where different species of oxidized Al and Si could be identified^37–39^.

In this work, we establish Auger electron photoelectron coincidence spectroscopy (APECS) as a powerful tool to determine chemical ESCA shifts previously inaccessible with the example of Ni 3*p* core-levels in coincidence with the *MVV* Auger electrons of a Ni(111) single crystal exposed to residual atmospheric gases at cryogenic temperature. Due to the coincidence technique, which allows to reduce the width of the ESCA spectrum by selecting specific final states^40^, the 3*p* core-levels could be narrowed to 1.2 eV FWHM. Thus the spin–orbit splitting of 1.6 eV could be resolved and a chemical shift of 0.6 eV determined for the Ni 3*p* levels of a Ni(111) single crystal and its oxidized surface layer.

Figure 1 depicts a schematic of the coincidence technique and energy levels to assist the comprehension of the experiment and the presented data. As shown the photoionization and the corresponding decay can take place either on the bulk atoms or the surface Ni atoms leading to different spectral features. Selecting specific Auger channels measured in coincidence with the Ni 3*p* photoelectrons we disentangled the photoelectron peaks corresponding to the bulk or the surface component, which are chemically shifted against each other.

## Methods

In an APECS experiment Auger- and photoelectrons originating from the same ionization event are measured simultaneously using two or more electron spectrometers. The data was obtained at the CoESCA end-station^41^ at the BESSY II UE-52 PGM beamline utilizing the Pulse Picking by Resonant Excitation method^42^ during multi-bunch operation. The main chamber is equipped with two angular resolved time-of-flight spectrometers (ArTOF)^43,44^. Those are well suited for electron–electron coincidence measurement, since this detector type provides a good timing resolution^45,46^, high transmission and allows for single shot computation. Thus, for a coincidence experiment no additional setup is required, since the same trigger synchronized with the X-ray pulses is used for both spectrometers. For the APECS measurement, the photon excitation energy was set to $h\nu =250\,{\text {eV}}$ with horizontal polarization, where one ArTOF was set to detect Ni 3*p* photoelectrons, and the second to detect the *MVV* Auger electrons. One spectrometer was aligned to detect electrons in a 176–183 eV kinetic energy window, which covers the full 3*p* photoelectron spectrum. The second spectrometer was aligned to detect electrons in a 53–60 eV kinetic energy window, which covers partly the *MVV* Auger electrons. For the calibration of the energy scale tabulated values were used^17,47^. In both cases the energy scales are referenced to the Fermi level. The total experimental energy resolution for both spectra is about 0.3 eV.

The raw data in a coincidence measurement contain both true and accidental counts^48–50^. One true count consists of one photoelectron and one Auger electron, connected to a single photoionization event. The accidental counts correspond to electrons from different photoionization processes and thus from two different atoms, which accidentally arrive in the same time window on the two detectors.The ratio between true and accidental coincidences sets a limit on what intensities are practical to use. Since the number of the true countrate is proportional to the incoming light intensity *I* and the accidental countrate is proportional to $I^2$, it is usually desirable to measure at low intensities despite the increased measurement time. Anyway, since only true counts reflect the real behaviour of the system the raw data has to be corrected. For the correction we used data from two consecutive synchrotron pulses as a measure for the accidental intensity. This allows to generate a dataset containing only accidentals measured under identical conditions. Subtracting the accidental counts from the raw data counts leads to the true coincidence dataset. For a detailed explanation of the APECS technique and the setup used for the presented experiment see Leitner et al.^41^. In the presented experiment the raw map contains 71,075 counts, the accidental map 53,187 counts and the true map 17,889 counts. The data acquisition time amounts to 28 h. Note, that the single, non-coincident Auger electron or photoelectron spectra can be extracted from the same dataset.

Due to the simultaneous measurement additional information can be gained from the dataset, besides the non-coincident Auger and photoelectron spectrum. By selecting only specific regions in the Auger spectrum one can select particular Auger final states and observe exclusively the corresponding initial core-hole states in the corresponding photoelectron spectrum. The other way around, selecting specific photoelectrons regions means that only particular initial states contribute to the resulting Auger spectrum. This is particularly useful to gain information about specific core-hole relaxation pathways^31^, but also for chemical analysis especially in the case of overlapping features.

The main chamber was operated under UHV conditions ($10^{-10}\,{\text {mbar}}$). The Ni(111) single crystal was cleaned in the preparation chamber ($10^{-10}\,{\text {mbar}}$) by heat treatment at 520 K for $24\,{\text {h}}$, in order to degas the sample and subsequently treated by several sputtering (Ar+ $5\cdot 10^{-6}\,{\text {mbar}}$, $20\, {\text {min}}$) and annealing (1070 K, $5\,{\text {min}}$) cycles. The purity was ensured by overview PES measurements, which showed only small traces of O and C. Afterward, the sample was exposed to air at $10^{-7}$ mbar and cooled down during the exposure using liquid Helium below 80 K. The process yields a large O 1*s*, O 2*s* and O Auger peak. Additionally, traces of C and N are present on the surface.

## Results

**Figure 2:**
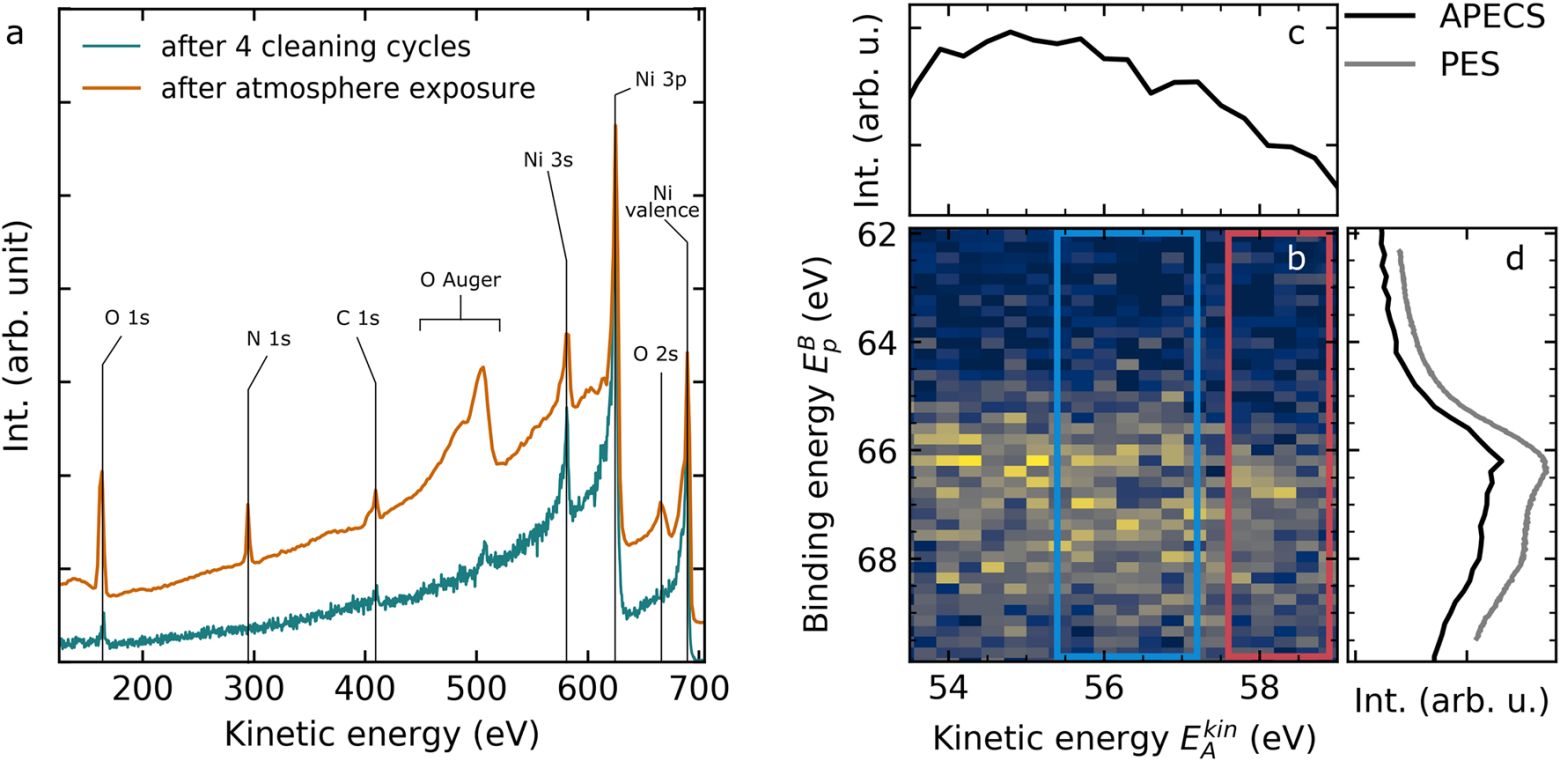
(**a**) PES overview spectra of Ni(111) obtained at 700 eV photon energy. In cyan a PES survey of Ni(111) after multiple cleaning cycles is shown. In orange a PES survey of the air exposed Ni(111) is shown. The spectra were normalized to the Ni 3*p* intensity. An offset was introduced for better visibility. Peaks are assigned to the corresponding elements. (**b**) Ni *MVV* Auger electron ($E_{Au}$) Ni 3*p* photoelectron ($E_{pe}$) coincidence map (17,889 true counts) corrected for accidental counts. The marked regions are used for later analysis shown in Fig. 3. (**c**) Integration of the full map along the photoelectrons ( along the y-axis, 62–70 eV binding energy) results in *MVV* Auger spectrum. (**d**) Integration of the full map along the Auger electron axis (along the x-axis, 53–60 eV kinetic energy) results in 3*p* photoelectron spectrum. In gray a non-coincidence ESCA spectrum is shown. The figure was created using the python matplotlib package^53^ and inkscape^54^.

Figure 2a shows an overview PES spectrum at 700 eV X-ray excitation energy. The features were assigned according to available XPS data^11,47,51,52^ The cyan spectrum shows Ni(111) after several cleaning cycles. Besides the expected Ni 3*p*, 3*s* and the valence band small residues mainly originating from O and C are present. The orange spectrum in Fig. 2a shows the Ni(111) sample after exposure to air. The C contamination slightly increased, and additionally a small amount of N on the surface appeared. The main change however is due to O at around 162 eV (O 1*s*), 666 eV (O 2*s*) and a large contribution from the O Auger decay (450–520 eV). A similar behaviour is observed for several preparation cycles, although the amount of N and C showed slight variations. Even though air has a large N amount the adsorption of $\hbox {N}_2$ on a Nickel surface is less likely then for O or $\hbox {O}_2$. This is, due to a very strong bonding between the N atoms.

Comparison of the O Auger region with tabulated Auger energies by W. E. Moddeman et al.^51^ indicates no ice formation is taking place on the surface, since $\hbox {H}_2$O should have a substantially narrower Auger spectrum and does not exhibit features above 500 eV. Even though, we cannot completely exclude the presence of $\hbox {H}_2$O the results indicate that the main contribution originates from molecular and atomic oxygen.

The APECS study was performed at cryogenic temperature. In this experiment we measure the kinetic energy of the photoelectrons and that of the Auger electrons in coincidence. However, it is more convenient to show the photoelectrons on the binding energy scale. For an oxidized Ni surface the 3*p* levels are shifted towards higher binding energies compared to clean Ni, while the Auger electrons are shifted towards higher kinetic energy (see Fig. 1). Hence, by selecting Auger electrons with higher kinetic energy in the dataset means that we are more sensitive to the 3*p* photoelectrons corresponding to oxidised Ni atoms, which are located on the surface. Figure 2b shows the Ni *MVV* Auger 3*p* photoelectron true coincidence map together with the spectra obtained by integration of the full map along the Auger kinetic energy (c) and along the photoelectron binding energy (d).

**Figure 3:**
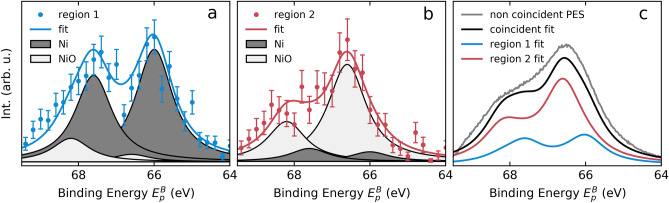
Ni 3*p* spectra in coincidence with different *MVV* Auger electron regions. (**a**) 3*p* spectrum in coincidence with the lower kinetic energy Auger region (Fig. 2b blue square). The final fit is shown as a solid blue line and contains the spin–orbit split $3p_{3/2}$ and $3p_{1/2}$ peaks from bulk Ni in dark gray and the spin–orbit split $3p_{3/2}$ and $3p_{1/2}$ contribution from the Ni surface in light gray. (**b**) 3*p* spectrum in coincidence with the higher kinetic energy Auger region (Fig. 2b red square). The final fit containing the same components as in (**a**) is shown as a solid red line. (**c**) In gray the non-coincidence PES Ni 3*p* measurement is shown together with a weighted sum (black) of the fits from (**a**) (blue) and (**b**) (red). The figure was created using the python matplotlib package^53^.

At first glance the full, coincident Ni *MVV* Auger spectrum (Fig. 2c) shows no characteristic features. This is due to the small energy window recorded, which only contains a part of the full 15 eV broad *MVV* Auger spectrum^17^. Also the Ni full, coincident 3*p* photoelectron spectrum (Fig. 2d) shows no distinct features, which is in agreement with already published measurements^10,11^. For comparison, a non-coincidence ESCA 3*p* spectrum measured under identical conditions is included as a gray, solid line. The non-coincident data were obtained from the same dataset as the coincidence data by analysing all counts in one spectrometer, disregarding the coincidence condition. The map (Fig. 2b) on the other hand shows two interesting features. One is the drop of intensity at about 57 eV kinetic energy and 67 eV binding energy (blue frame). The other feature is the photoelectron intensity shift towards higher binding energy at Auger energies above 57.5 eV (red frame). In order to analyze these features closely we selected two regions in the map. The integration along the Auger electron axis of the framed regions is shown in Fig. 3a,b using the same color code. Note, that a Shirley background has been subtracted from the shown data^14,55^. The 3*p* spectrum obtained in coincidence with the blue Auger region (Fig. 3a), shows two spin–orbit split peaks, i.e. the $3p_{3/2}$ and the $3p_{1/2}$ split by about 1.6 eV. This is in agreement with published results^10,47^, which report values of 1.7 eV or 1.8 eV, respectively. Before we discuss the fit model shown in the Fig. 3, we first have to consider the 3*p* spectrum obtained in coincidence with the red Auger region Fig. 2b. In the integrated spectrum shown in Fig. 3b we see a main peak at around 66.5 eV binding energy with a shoulder towards higher binding energy. Comparison of the spectra in Fig. 3a,b shows a chemical shift of the main peaks ($3p_{3/2}$) of about 0.6 eV related to the surface oxidation. In order to quantify the spectral components, we introduce a fit model consisting of four asymmetrical Voigt (skewed Gaussian convoluted with a Lorentzian profile) peaks with position, amplitude, skewness and width being free fit parameters. The width was set to be the same for all four peaks. With the previously gained knowledge we also fixed the spin–orbit splitting at 1.6 eV for both species. Thus, the model accounts for the Ni bulk and the surface oxidised species, as well as for the spin–orbit splitting. The spin–orbit split photoelectron peaks in dark gray originate from bulk Ni. The spin–orbit split photoelectron peaks in light gray originate from the oxidized Ni surface.

The final fits are in good agreement with the experimental data and are shown as a solid colored line in Fig. 3a,b. The resulting width of the individual peaks is 1.2 eV (FWHM), whereas the Gaussian width is set to 0.3 eV reflecting the experimental resolution. The Lorentzian width results in 1.1 eV and fits very closely to the published results by Nyholm et. al.^8^, who derived the Lorentzian width of the Ni 3*p* peaks (1.19 eV) using a broadened 2*p* spectrum.

The intensity of the individual peaks is rather counterintuitive e.g. the $3p_{1/2}$ peak is sometimes more intense than the $3p_{3/2}$ peak, which ratio should be 1/2 in a non-coincident photoelectron spectrum. In a photoelectron Auger electron coincidence experiment however, specific final states are selected when cutting the Auger region in Fig. 2b. These final states correspond to the decay of different core-holes ($p_{1/2}$ and $p_{3/2}$) and to the different chemical states of pure bulk Ni and the NiO surface. Thus, the selected regions include differently weighted contributions by the different final states (some final states are excluded completely), which causes the observed intensity redistributions in the corresponding photoelectron spectra. The possibility to obtain spectra with different relative intensities of the individual photoelectron peaks is a powerful tool when determining the positions of these strongly overlapping peaks. Selecting more Auger transitions, which correspond to the decay of $p_{1/2}$ core-hole ionised states lead to an enhanced $p_{1/2}$ intensity in the corresponding photoelectron spectra. In this experiment we have no means to identify the exact final states, due to the broad and overlapping multiplet structure in the Auger spectrum. However, only the peak positions are within the aim of the study and not the intensity ratios.

To evaluate our approach we compared the results with the non-coincidence Ni 3*p* ESCA spectrum. Figure 3c shows the non-coincidence 3*p* ESCA spectrum in gray. In blue and red, the fits of the bulk Ni and oxidized Ni surface are shown, respectively. By only adjusting the heights of these two contributions we could achieve good agreement with the experimental non-coincidence Ni 3*p* ESCA spectrum. The sum of the adjusted partial spectra is shown as a solid black line. Note, that the non-coincident spectrum is broader than the partial coincident spectra due to the life-time broadening but also due to inelastic background, which is reduced in the coincident experiment. Here the model serves only for demonstration purposes, but in principle it has to be corrected for some missing intensities.

## Discussion

We demonstrated the capabilities of APECS for the Ni 3*p* photoelectron spectrum. The Ni 3*p* ESCA spectrum is broad and featureless, which substantially complicates quantitative studies. Particularly, if the atomic environment causes energetic shifts leading to additional contributions in the spectrum, conventional methods reach their limits. As shown, the *MVV* Auger spectrum is broad and does not exhibit distinct features. Thus, it is very difficult to identify regions corresponding to specific final states. Nevertheless, by utilizing APECS we are able to narrow the photoelectron lines and deduce their widths and positions from the experimental data. This is due to a great advantage of the coincidence technique to be able to select specific decay channels^40,56^. In our case, this can be achieved by simply analysing selected regions in the 2D data map.

In an ideal APECS experiment the linewidth of the intermediate core-hole state should be completely removed from the spectra. Due to the width and the overlapping final state multiplet lines in the Auger spectrum, it is impossible to select specific final states. Thus, the width of the photoelectron peaks is strongly influenced by the selected Auger regions, since different final state multiplets or tails of some multiplet lines outside of the selected regions contribute to the width of the photoelectron lines. Therefore, our results do not reflect the real lifetime of the 3*p* core-holes. But, due to the narrowing effect of the method, we to our knowledge for the first time are able to observe different contributions to the 3*p* photoelectron spectrum and also deduce the peak positions and the chemical shifts. The analysis of the selected regions narrows the Ni 3*p* widths to 1.2 eV and gives access to the identification of two different 3*p* species separated by a chemical shift of 0.6 eV. The obtained results are robust against parameter or shape variations and the errors are within $\pm 0.1$ eV.

We performed a PES survey, which indicates that the main contribution to the chemical shift is due to the native oxide layer on the Ni surface. The results are in agreement with McIntyre et al.^11^, who reported a Ni $3p_{3/2}$ chemical shift in NiO to be about 1 eV towards higher binding energies. This also fits together with the observation of the shift in coincidence with the higher kinetic energy Auger region (see Fig. 1), although we have no means to quantify the Auger spectrum. Finally, we confirm our observations and model by fitting the singles 3*p* ESCA spectrum using the line shapes obtained from the analysis of the APECS data. Comparison of the intensities of the two contributions shows that the oxidized Ni contributes more to the 3*p* spectrum than the pure Ni, demonstrating the surface sensitivity of the experiment. Calculating the effective inelastic mean free path for this particular coincidence experiment^14^ yields about 0.3 nm, which is close to the lattice parameter of Ni^57^. This indicates that in this experiment we are sensitive to the first two atom layers namely the contaminated surface layer and the pure Ni bulk component.

## Conclusions

In conclusion we show how Auger electron photoelectron coincidence spectroscopy (APECS) allows for separation of chemically shifted species inseparable in classical ESCA.

APECS at the Ni *M*-edges of a Ni(111) single crystal exposed to residual gas made it possible to extract the chemical shifts in the photoelectron spectrum by measuring in coincidence with different regions of the Auger spectrum. In this way we could identify and quantify different species corresponding to the oxidized Ni surface layer on a bulk Ni(111) crystal. In this study we also made use of the enhanced surface sensitivity of APECS. With a simple model we have reconstructed the conventional Ni 3*p* ESCA by using the APECS data confirming that all essential chemical states are captured in our analysis but going far beyond the limitations of conventional PES.
